# Supplementary material for: Structure of a thylakoid-anchored contractile injection system in multicellular cyanobacteria
Source: Nat Microbiol. 2022 Feb 14;7(3):386–96. doi: 10.1038/s41564-021-01055-y (PMC8894136; doi:10.1038/s41564-021-01055-y)
Supplement: Supplementary file 1 — Supplementary Figs. 1–11, Tables 1–9, legends of Videos 1–4, source data. [file 41564_2021_1055_MOESM1_ESM.pdf]

nature portfolio

https://doi.org/10.1038/s41564-021-01055-y

Supplementary information

Structure of a thylakoid-anchored contractile injection system in multicellular cyanobacteria

In the format provided by the authors and unedited

Supplementary Information

Supplementary figures 1 – 11

Supplementary tables 1 – 9

Supplementary movies 1 – 4

Supplementary source data

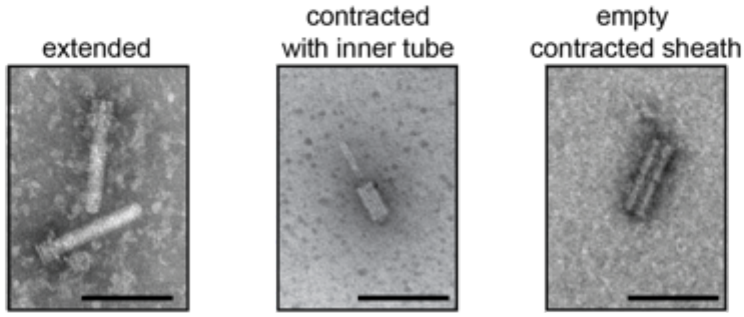

b

locus tag	gene name	unique peptide counts
alr3311	-	20
alr3312	-	2
all3314	*cis19*	60
all3315	*cis12*	59
all3316	*cis13*	5
all3317	*cis11*	50
all3318	*cis9*	6
all3320	*cis8*	15
all3323	*cis5*	7
all3324	*cis1*	14
all3325	*cis2*	24
all3326	*cis16B*	2
all3327	*cis16A*	6

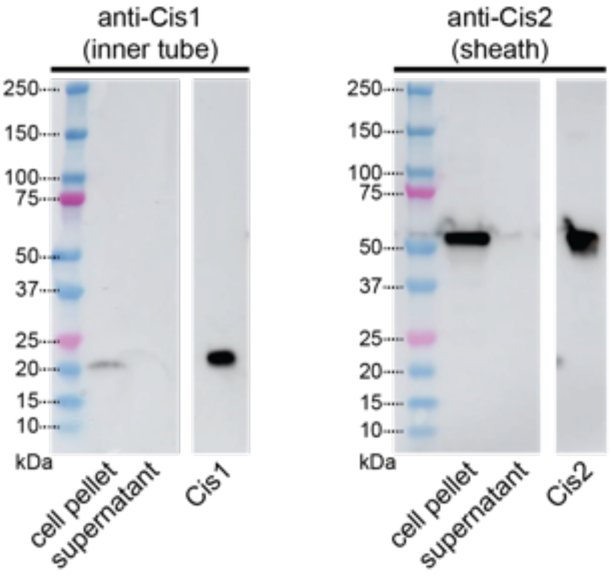

Supplementary Figure 1: CISs of *Anabaena* are expressed, but not released into culture supernatant.

a: Negative-stain electron micrographs of CISs in different conformations. The experiment was repeated eight times with similar results. Bars, 100 nm.

b: Proteins identified by mass spectrometry of purified CISs.

c: Western blots of cell pellet and precipitated culture supernatant showed that inner tube (Cis1) and sheath (Cis2) proteins were localized in cells and were not released into the culture supernatant. The experiment was repeated three times with similar results.

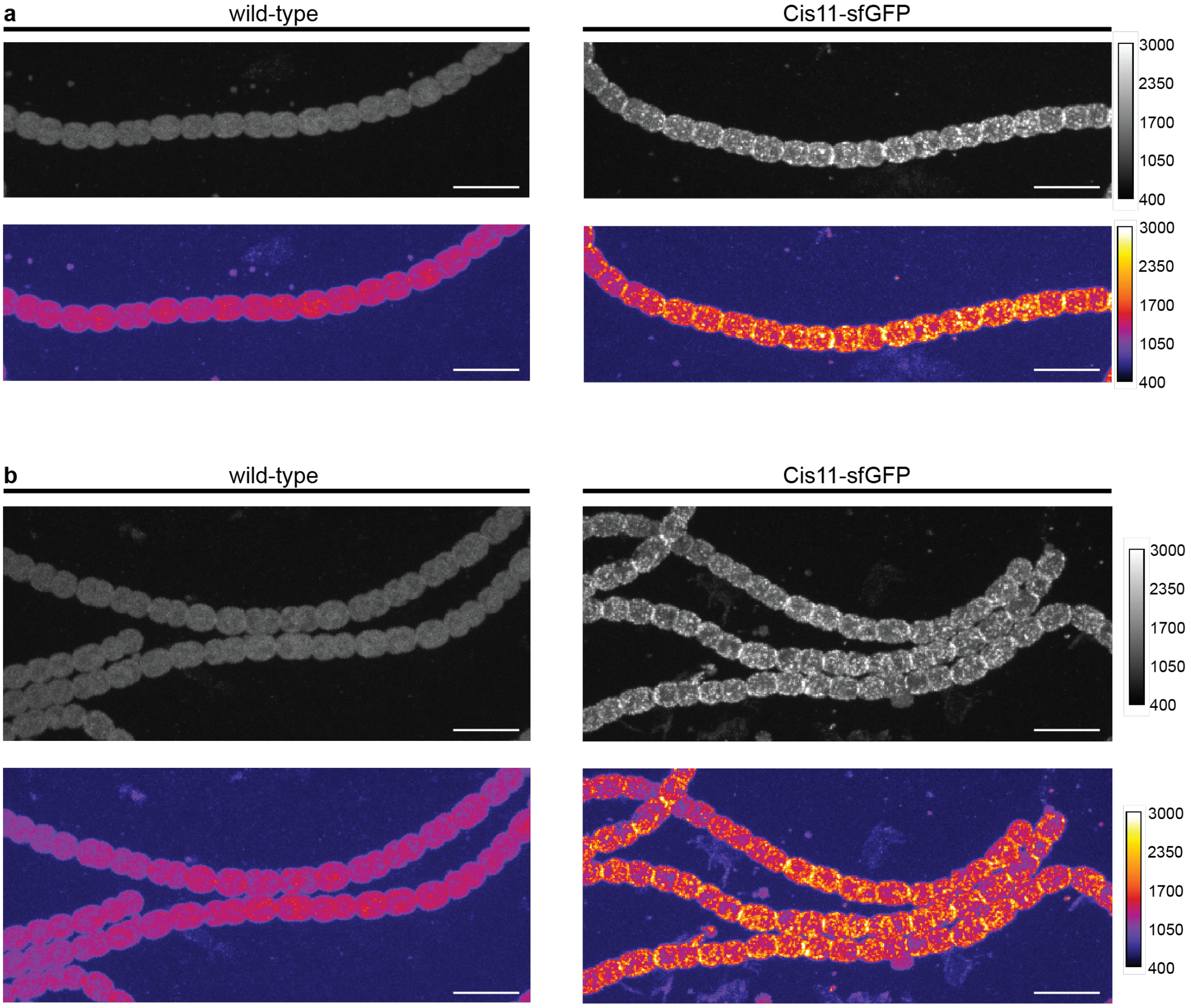

Supplementary Figure 2: GFP-foci are exclusively observed in *Anabaena* Cis11-sfGFP

a/b: Shown are two examples (a/b) of fLM images acquired at 488 nm excitation wavelength using a Nikon Eclipse T1 microscope equipped with a spinning disk module (Visitron). Discrete foci were only observed in *Anabaena* expressing Cis11-sfGFP (right panel) whereas no foci could be observed in *Anabaena* wild-type. Shown are grey-scale images (upper panel in a/b) and the same image in FIRE lookup table (LUT, lower panel in a/b) of maximum intensity projections. All images were acquired using identical imaging parameters and were normalized to same contrast/brightness values. Intensity calibration bar is shown on the right. The experiment was repeated four times with similar results and in total 29 confocal z-stacks were acquired. Bar, 10 µm.

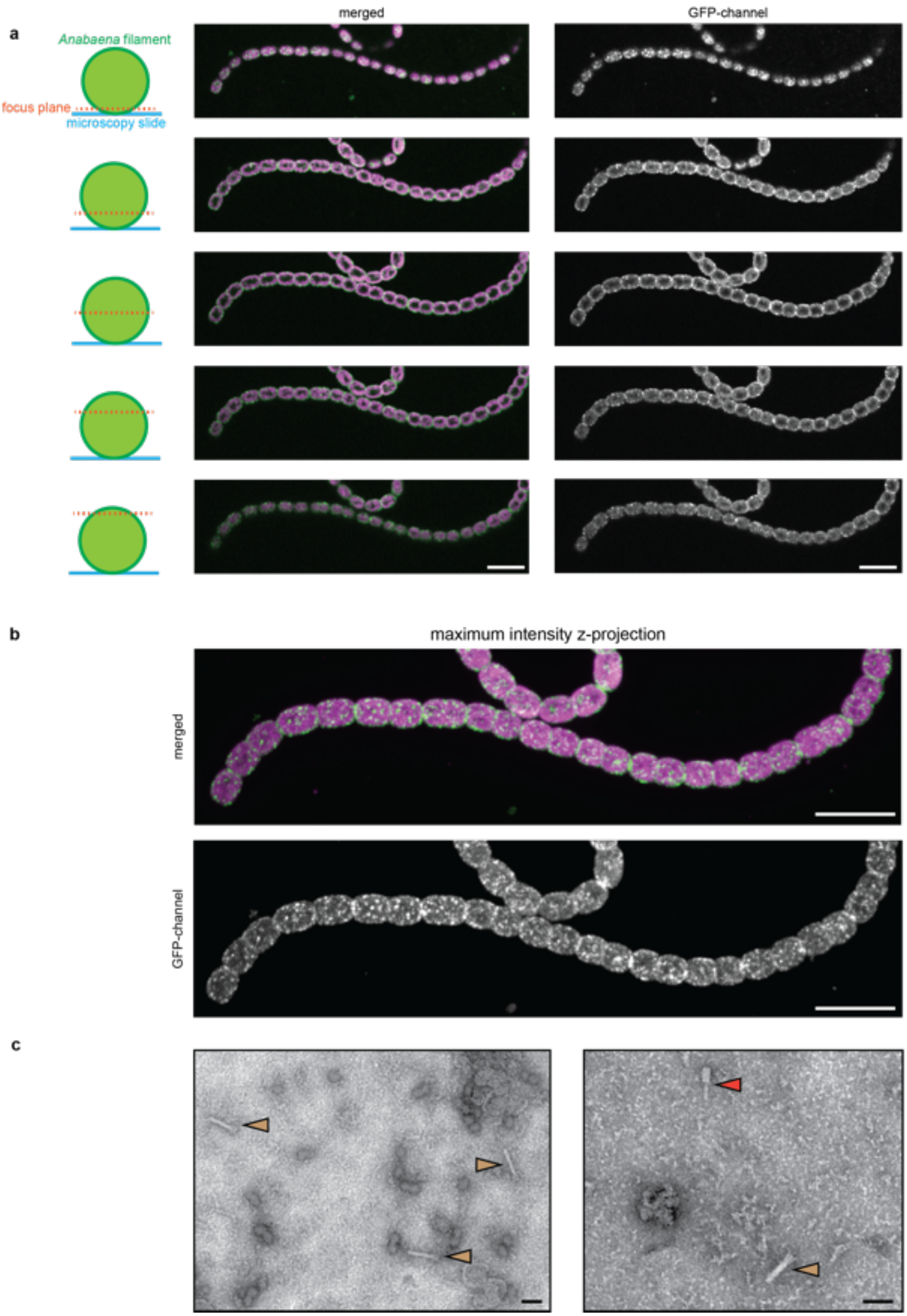
a
merged
GFP-channel
Anabaena filament
focus plane
microscopy slide
b
maximum intensity z-projection
merged
GFP-channel
c

27

Supplementary Figure 3: *Anabaena* expressing Cis11-sfGFP reveals numerous foci located in the periphery of the cell.

a: Confocal fLM Images of same *Anabaena* filament as shown in Fig. 1a at different focal planes (schematic on the left). GFP-foci (green) were mainly found in the periphery of the cell. Autofluorescence from chlorophyll is shown in magenta (left). A faint autoflourescence is detectable in the green channel together with GFP foci (right). The experiment was repeated four times with similar results and in total 29 confocal z-stacks were acquired. Bar, 10 µm.

b: Maximum intensity z-projection of all focal planes. Bar, 10 µm.

c: Negative-stain electron micrographs of CIS purified from *Anabaena* Cis11-sfGFP revealed fully assembled CIS in extended (brown arrowheads) and contracted conformation (red arrowhead). The experiment was repeated three times with similar results. Bars, 100 nm.

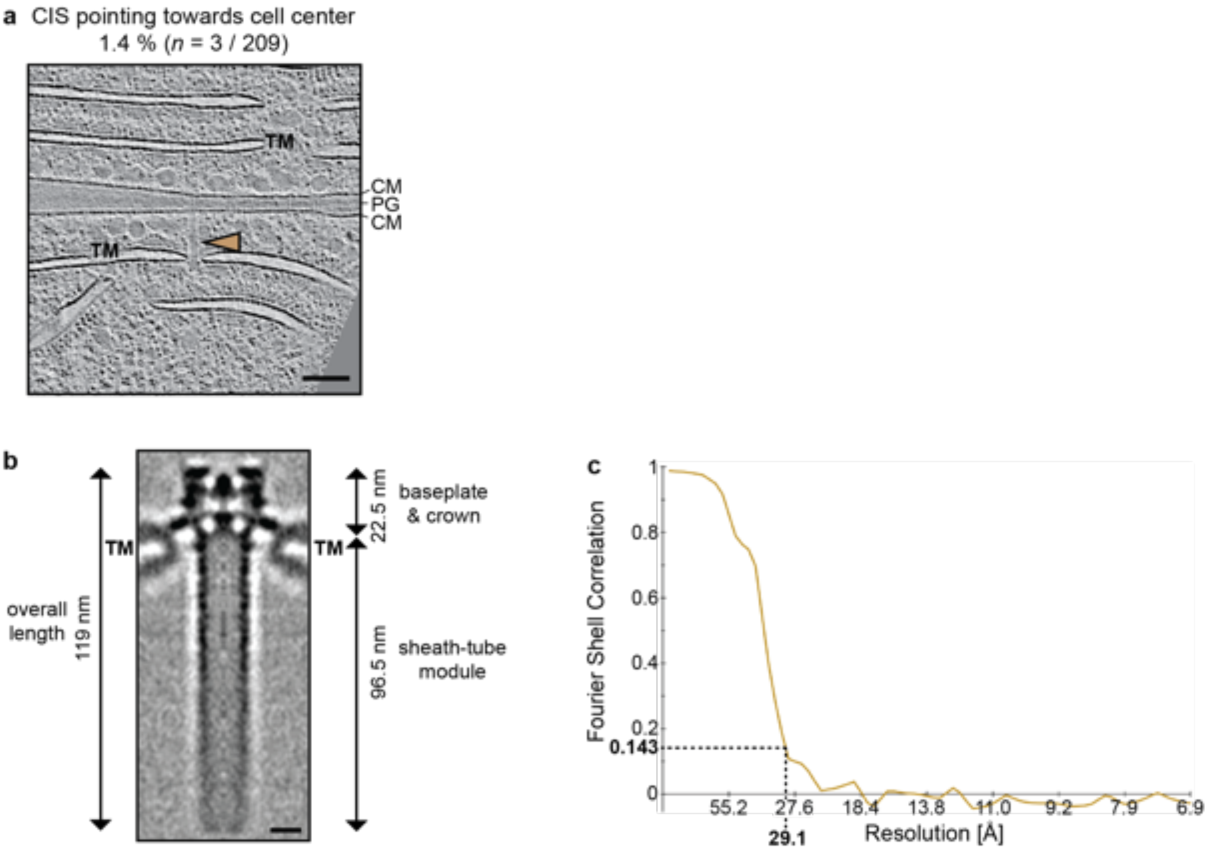

Supplementary Figure 4: *In situ* architecture of CISs in Anabaena

a: Slice through cryo-tomogram of cryoFIB-milled *Anabaena* cells. Whereas the vast majority of CISs pointed to the cell periphery, a low percentage (1.4%) of CISs (brown arrowhead) were facing the cell center (observed in three tomograms acquired in three independent experiments). CM, cytoplasmic membrane; PG, peptidoglycan; TM, thylakoid membrane stack. Shown is a 13.4 nm thick slice. Bar, 100 nm.

b: *In situ* subtomogram average (*n* = 204 particles from 99 tomograms acquired in 14 independent datasets, 6-fold symmetrized) of entire CIS apparatus revealed a thylakoid-anchored baseplate and a sheath-tube module, which is capped at the distal end. TM, thylakoid membrane stack. Bar, 10 nm.

c: Fourier Shell Correlation (FSC) analysis of two half-datasets of subtomogram average of CIS baseplate (shown in Fig. 1h) within intact *Anabaena* filaments resulted in approximate resolution of 29 Å.

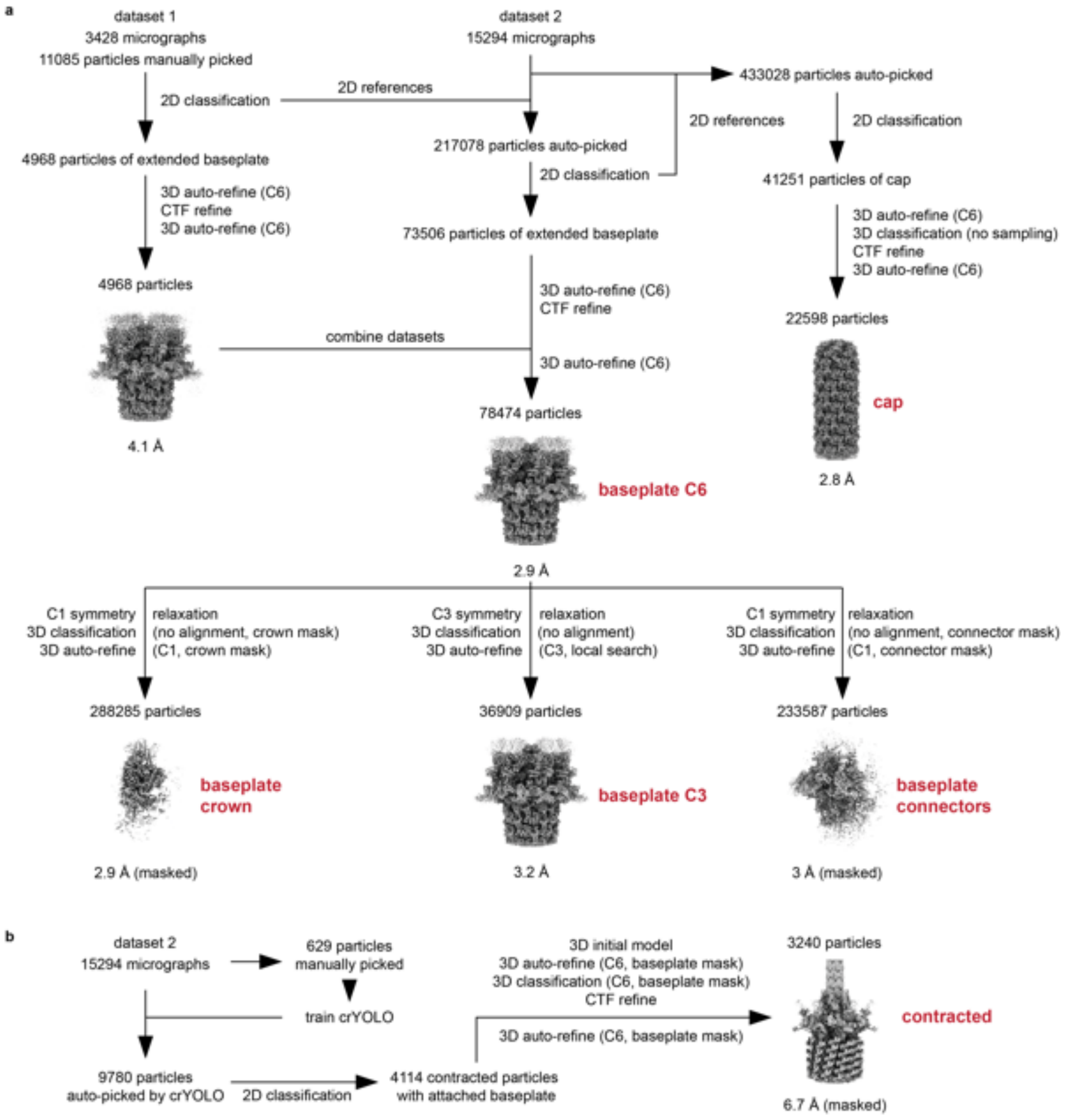

Supplementary Figure 5: SPA processing workflow

Shown are schematics of processing workflows used to obtain high-resolution maps of extended CIS (a) and contracted CIS (b).

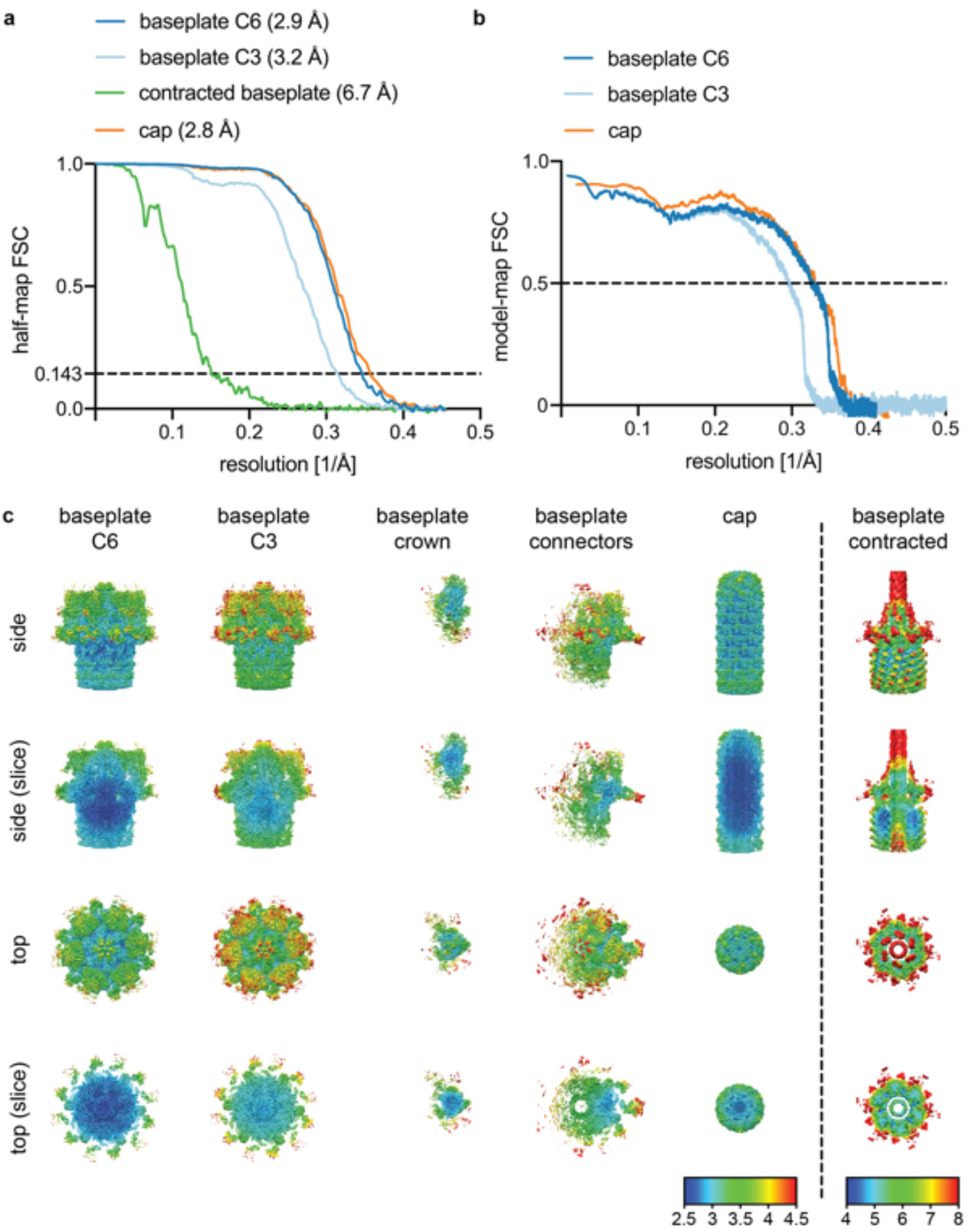

Supplementary Figure 6: SPA of CIS yield high-resolution structures.

a: Fourier shell correlation (FSC) plots of obtained maps generated from independent half data sets. Resolution was determined at a cut-off of 0.143.

b: FSCs of built atomic models and maps.

c: Local resolution distribution of all maps used for model building. Color scales in Å.

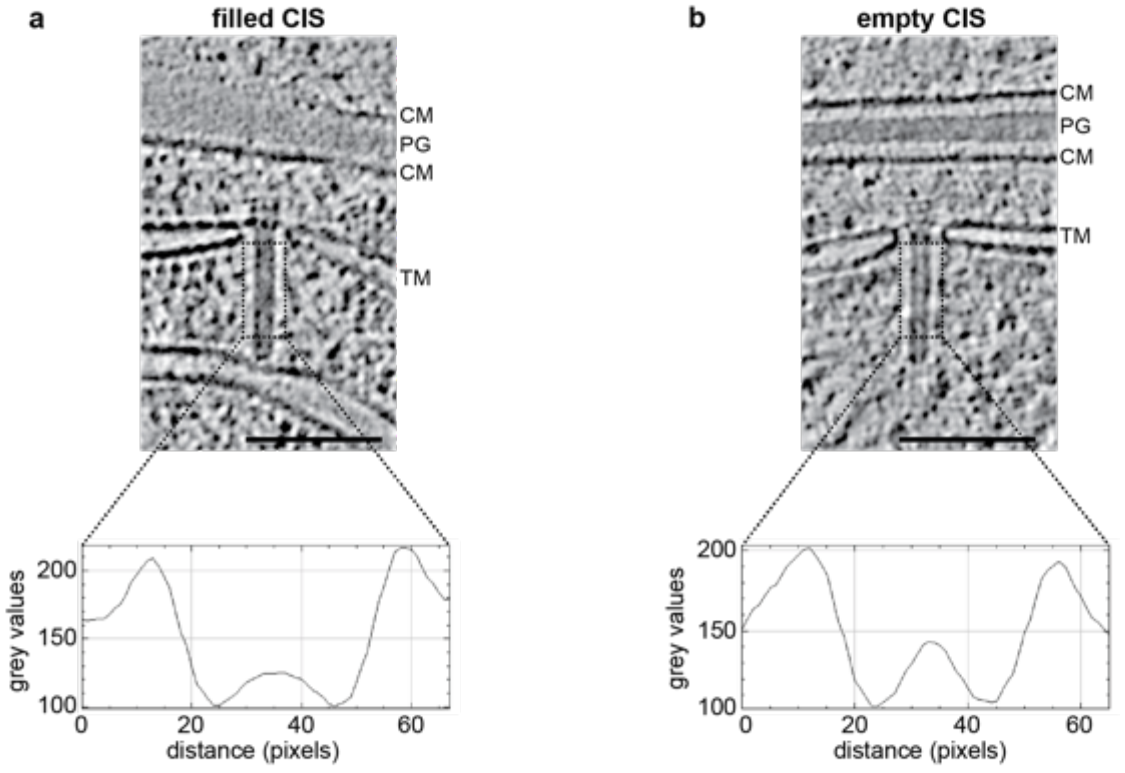

Supplementary Figure 7: CIS cargo might be loaded into tube lumen.

a: Slice through cryo-tomogram of filled CISs (n_{observed} = 204; shown is 13.5 nm thick slice). The area indicated by dashed box was used to calculate the density plot below. Bar, 100 nm.

b: Slice through cryo-tomogram of empty CISs (n_{observed} = 15; shown is 13.5 nm thick slice). The area indicated by dashed box was used to calculate the density plot below. The observation of empty CISs might indicate that potential cargo could be loaded into tube lumen. Bar, 100 nm.

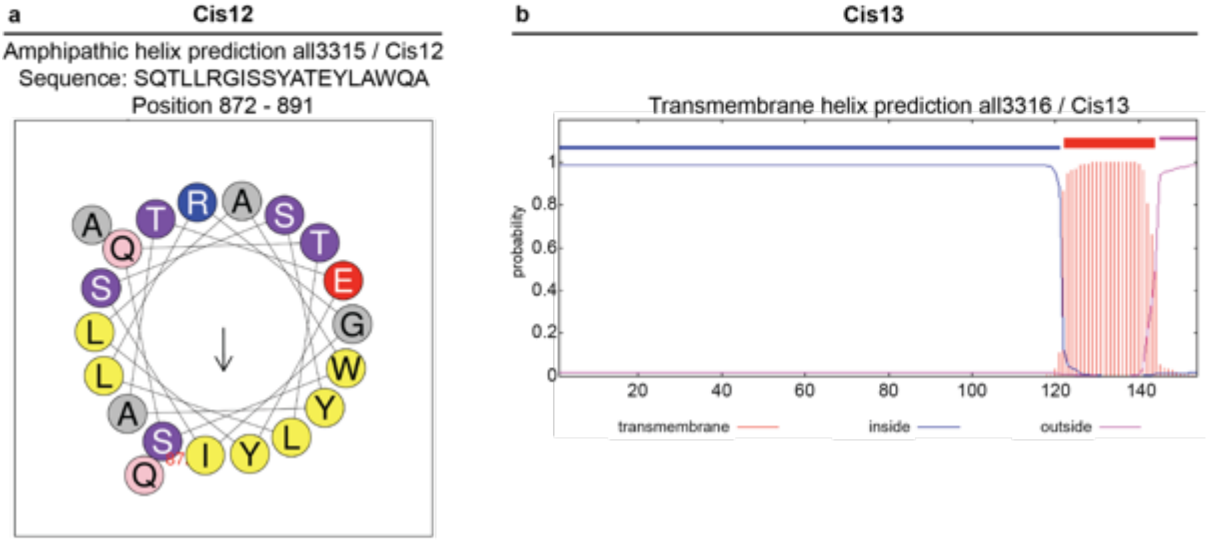

Supplementary Figure 8: Cis12 harbors predicted amphipathic helix whereas Cis13 contains putative transmembrane domain.

a: Shown is a schematic of the predicted amphipathic helix in Cis12 at position 872-891.

b: Shown is a schematic of the predicted transmembrane domain in Cis13.

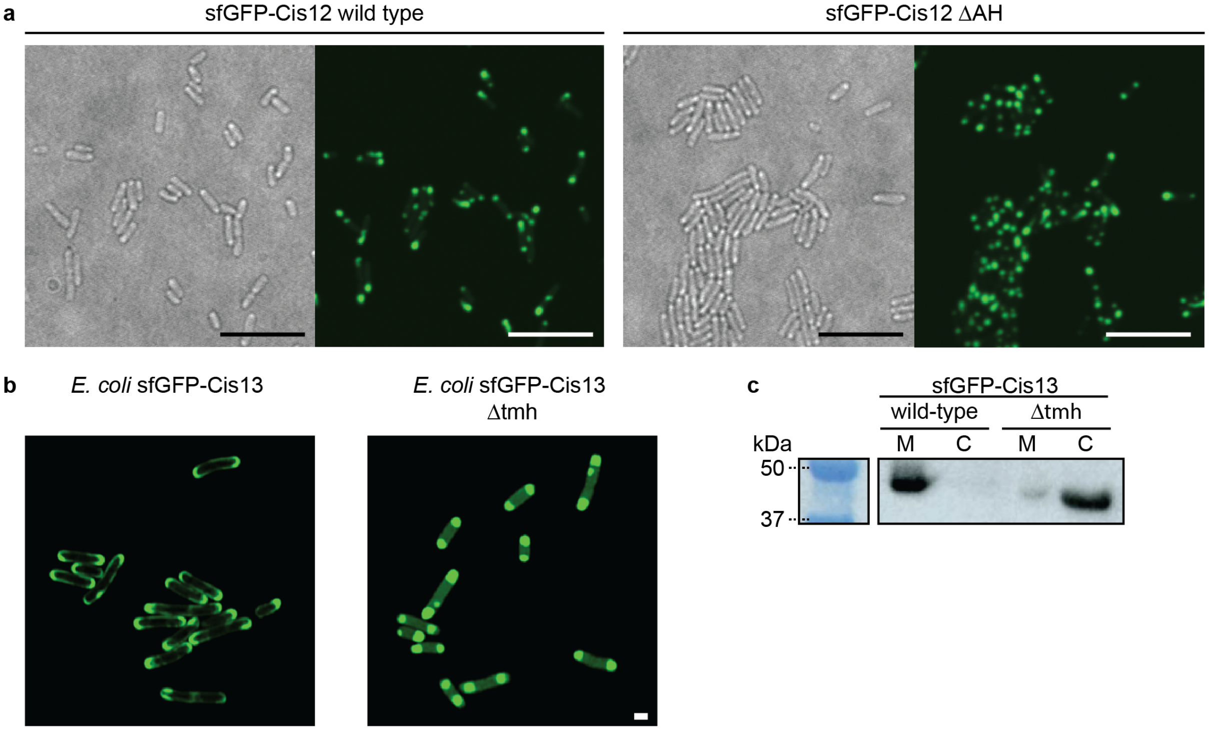

Supplementary Figure 9: Heterologously expressed sfGFP-Cis13 shows transmembrane helix dependent membrane association.

a: *E. coli* expressing full-length (wild-type) and truncated (ΔAH, missing peripheral domain containing amphipathic helix) sfGFP-fused Cis12 showed no association with the membrane. Large foci might result from sfGFP-Cis12 aggregates. Seven micrographs were collected for each sample. Bars, 10 µm.

b: Shown are fLM images of *E. coli* cells expressing full-length (wild-type) and truncated (Δtmh) sfGFP-Cis13. While the wild-type protein associated with the cell envelope, the transmembrane deletion mutant was dispersed in the cytoplasm and showed large foci that might correspond to aggregation. Eleven micrographs were collected for each sample. The experiment was repeated twice with similar results. Bars, 1 µm.

c: Western blotting of the membrane (M) and soluble (C) fractions from *E. coli* cells expressing full-length (wild-type) and truncated (Δtmh) sfGFP-Cis13 confirmed the membrane localization of the wild-type protein. Western blotting was not repeated.

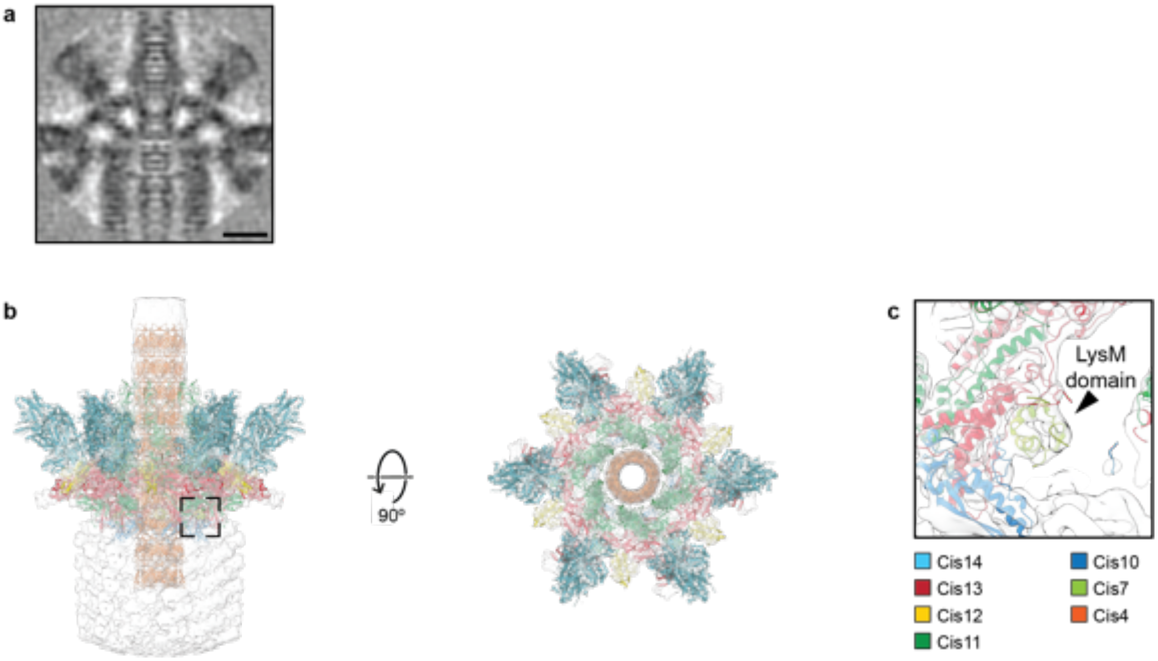

Supplementary Figure 10: Architecture of contracted CISs reveals the fate of crown and cage.

a: *In situ* subtomogram average showing contracted CIS still connected to TM with an opened crown conformation. Due to low particle numbers (n = 11), the average remained at low resolution (not estimated). Shown is a 4.3 nm thick slice. Bar, 10 nm.

b: SPA map of contracted CIS. The existing cryoEM dataset from purified CISs, which displayed a mixed population of extended and contracted conformations (about 11 % contracted CISs) was used for SPA processing (Extended Data Fig. S4). The final map at a resolution of 6.7 Å for the contracted baseplate complex (transparent, see Extended Data Fig. S4/S5 for reconstruction details) allowed rigid-body fitting of previously built atomic models from extended CIS structure (color code according to Fig. 2). Dashed box indicates magnified view in (c).

c: An additional density in the contracted baseplate map could be identified as the LysM domain of the tube initiator Cis7 that was cleaved and remained with the baseplate complex upon contraction while spike and tube initiators were expelled (see Extended Data Fig. S9 for extended state).

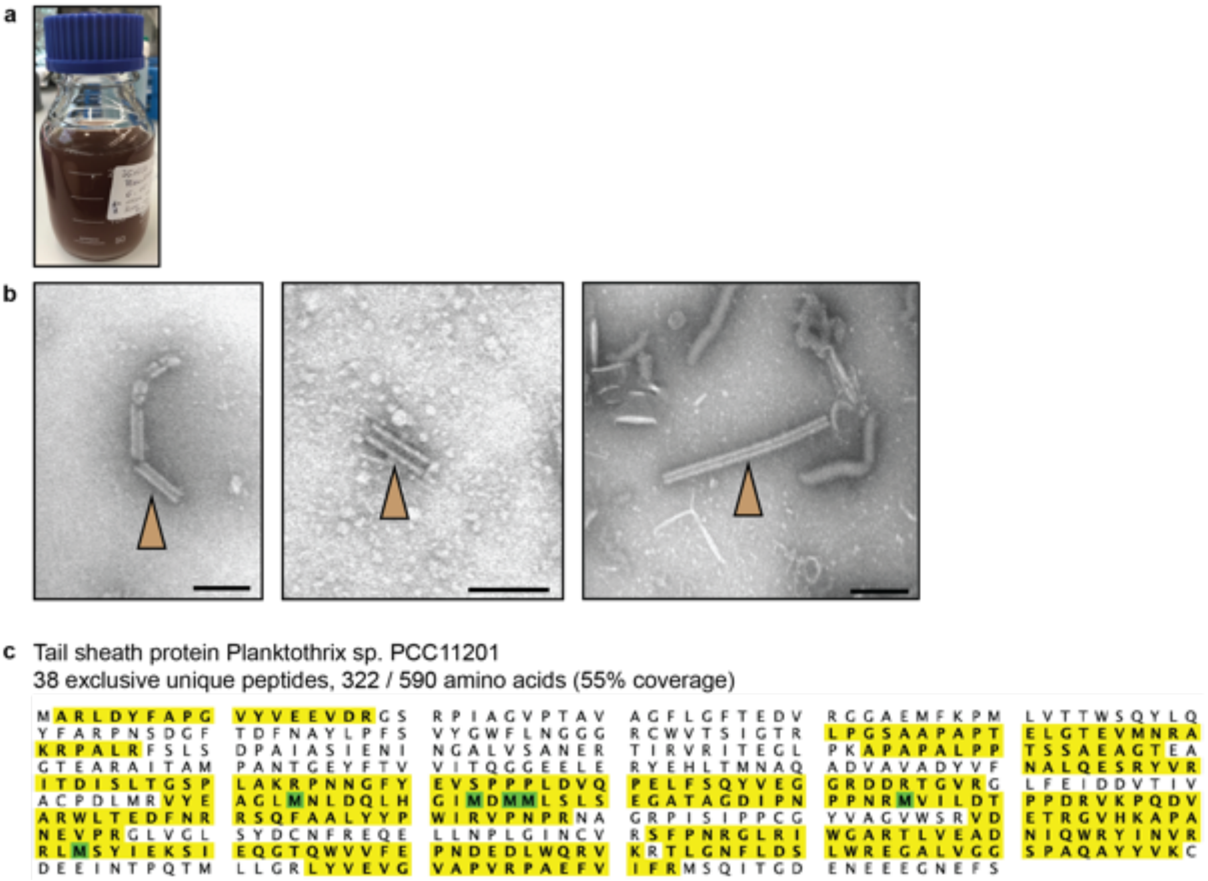

Supplementary Figure 11: CISs from *Cyanobacteria* can be readily found in the environment.

a: *Cyanobacteria*-enriched sample collected from Lake Zürich.

b: Three examples of negatively stained EM images of contracted sheath obtained from the sample shown in (a). Bars, 100 nm.

c: MS identified putative sheath protein from *Planktothrix* sp. PCC 11201 with 38 unique peptide counts and 55% sequence coverage (yellow; green, oxidation events). The experiment was not repeated.

Supplementary table 1: *Anabaena* CIS components compared with components of other CIS.

Anabaena	*S, entomophila*	*Photorhabdus*	*P. luteoviolacea*	T4 phage	T6SS	$T6SS^{IV}$	R-type pyocin	Putative function
Cis1	Afp1	Pvc1	JF50_12680	gp19	TssD	Aasi_1077	PA0623	inner tube
Cis2	Afp2	Pvc2	JF50_12675	gp18	TssB/C	Aasi_1074	PA0622	sheath
-	Afp3	Pvc3	-	gp18	TssB/C	-	-	sheath
-	Afp4	Pvc4	-	gp18	TssB/C	-	-	sheath
Cis5	Afp5	Pvc5	JF50_12685	gp54	-		-	tube initiator
Cis6	Afp6	Pvc6	JF50_12690?	-	-	Aasi_1078	-	spike plug
Cis7	Afp7	Pvc7	JF50_12695	gp48 (gp53-LysM)	-	Aasi_1079	PA00626, PA0627 (LysM)	tube initiator
Cis8	Afp8	Pvc8	VgrG	gp27/5	VgrG	Aasi_1080	PA0628/0616	spike
Cis9	Afp9	Pvc9	JF50_12705	gp25	TssE	Aasi_1082	PA0617	baseplate sheath adapter
Cis10	Afp10	Pvc10		gp5.4	PAAR	Aasi_1081	-	spike tip
Cis11	Afp11	Pvc11	JF50_12710	gp6/7	TssF/G	Aasi_0557	PA0618/0619	baseplate wedge (inner)
Cis12	Afp12	Pvc12	JF50_12715	gp6	TssF	Aasi_1083	PA0618	baseplate wedge (outer)
Cis13	Afp13	Pvc13	JF50_12720/12580	gp9/10/11/12	-	Aasi_0556	PA0620	tail fiber
Cis14	Afp14	Pvc14	JF50_12725	gp29	-	Aasi_1806	PA0625	tape measure
	Afp15	Pvc15	JF50_12570	-	(ClpV)		-	AAA+ ATPase
Cis16A/B	Afp16	Pvc16	JF50_12575	gp15	(TssA)	Aasi_1072	PA0615	cap

Supplementary table 2: CIS features are conserved in cyanobacterial genomes. Shown are sequence identities between *Anabaena* proteins and homologs in other cyanobacterial strains obtained with blastp. Strains not identified in Chen et al., 2019[16] were marked with asterisk.

**Strain (morphology)**	**crown (Cis19)**	**baseplate extension (Cis12:610-1100)**	**trans-membrane (Cis13:122-154)**
Anabaena sp. PCC 7120 (filamentous)	100 %	100 %	100 %
Trichormus variabilis NIES-23 (filamentous)	100 %	100 %	100 %
Fremyella diplosiphon NIES-3275 (filamentous)	52 %	45 %	52 %
Tolypothrix tenuis PCC 7101 (filamentous)	53 %	44 %	52 %
Aulosira laxa NIES-50 (filamentous)	53 %	44 %	52 %
Nostoc carneum NIES-2107 (filamentous)	52 %	45 %	52 %
Calothrix sp. PCC 6303 (filamentous)	43 %	40 %	-
Calothrix sp. NIES-3974 (filamentous)	44 %	42 %	-
Calothrix sp. 336/3 (filamentous)	43 %	37 %	-
Scytonema sp. UIC 10036 (*) (filamentous)	67 %	62 %	77 %
Leptolyngbyaceae cyanobacterium RU_5_1 (*) (filamentous)	55 %	56 %	68 %
Synechococcus sp. PCC 7336 (*) (unicellular)	34 %	39 %	-

Supplementary table 3: Experimental approaches to detect effects of *Anabaena* CIS on predatory organisms.

Target organism	Functional assay	Procedure
Amoebae	Killing assay on plate and in liquid	Co-incubation with *Anabaena* wild-type and CIS^{-} mutant strain<br>Co-incubation with purified CISs from *Anabaena*
Ciliates	Feeding	Feeding of *Anabaena* wild-type and CIS^{-} mutant strain to ciliate cultures
Daphniae	Feeding	Feeding of *Anabaena* wild-type and CIS^{-} mutant strain to Daphnia cultures
Insect cells	Killing assay in liquid	Co-incubation with *Anabaena* wild-type and CIS^{-} mutant strain
Hydroides elegans tubeworm larvae	Metamorphosis assay	Co-incubation with *Anabaena* wild-type and CIS^{-} mutant strain
Wax moth larvae	Injection into larvae gut	Injection of *Anabaena* wild-type and CIS^{-} mutant strain<br>Injection of purified CISs from *Anabaena*

Supplementary table 4: Experimental approaches to detect effects of *Anabaena* CIS on interacting organisms.

Target organism	Functional assay	Procedure
Acanthamoeba castellanii 5a2	Killing assay on plate and in liquid	Co-incubation with UV treated *Anabaena* wild-type and CIS^{-} mutant strain<br>Co-incubation with purified CISs from *Anabaena*
Anabaena PCC 7120	Killing assay on plate	Co-incubation with UV treated *Anabaena* wild-type, Cis13-Δtmh and CIS^{-} mutant strain
Candida albicans	Killing assay on plate	Co-incubation with *Anabaena* wild-type and CIS^{-} mutant strain<br>Co-incubation with UV treated *Anabaena* wild-type and CIS^{-} mutant strain
E. coli DH5a	Killing assay on plate	Co-incubation with *Anabaena* wild type and CIS^{-} mutant strain<br>Co-incubation with UV treated *Anabaena* wild-type and CIS^{-} mutant strain
Pseudomonas fluorescence	Killing assay on plate	Co-incubation with *Anabaena* wild-type and CIS^{-} mutant strain<br>Co-incubation with UV treated *Anabaena* wild-type and CIS^{-} mutant strain
Pseudomonas aeruginosa	Killing assay on plate	Co-incubation with *Anabaena* wild type and CIS^{-} mutant strain<br>Co-incubation with UV treated *Anabaena* wild-type and CIS^{-} mutant strain
Sf9 insect cells	Killing assay in liquid	Co-incubation with UV treated *Anabaena* wild-type and CIS^{-} mutant strain
Staphylococcus saprophyticus	Killing assay on plate	Co-incubation with *Anabaena* wild-type and CIS^{-} mutant strain<br>Co-incubation with UV treated *Anabaena* wild-type and CIS^{-} mutant strain
Serratia entomophila	Killing assay on plate	Co-incubation with *Anabaena* wild-type and CIS^{-} mutant strain<br>Co-incubation with UV treated *Anabaena* wild-type and CIS^{-} mutant strain

Serratia protemaculans	Killing assay on plate	Co-incubation with *Anabaena* wild-type and CIS^{-} mutant strain<br>Co-incubation with UV treated *Anabaena* wild-type and CIS^{-} mutant strain
Synechococcus PCC 7942	Killing assay on plate	Co-incubation with *Anabaena* wild-type and CIS^{-} mutant strain<br>Co-incubation with UV treated *Anabaena* wild-type and CIS^{-} mutant strain
Synechocystis PCC 6803	Killing assay on plate	Co-incubation with *Anabaena* wild-type and CIS^{-} mutant strain<br>Co-incubation with UV treated *Anabaena* wild-type and CIS^{-} mutant strain

Supplementary table 5: Strains and plasmids used in this study.

Strain	Relevant characteristic	Reference
Anabaena sp. PCC7120	wild-type	Rippka et al., 1979[32]
Anabaena sp. PCC7120 Cis11-sfGFP	Cis11-sfGFP, Nm^r, Sm^r, Sp^r	This study
Anabaena sp. PCC7120 DR815	Cis13-Δtmh, Nm^r	This study
Anabaena sp. PCC7120 CIS^{-}	*cis2*::pRL277, Sm^r, Sp^r	This study
E. coli BL21Star™ (DE3)		Invitrogen
E. coli NEB 10β		NEB

Plasmid	Relevant characteristics	Reference
pET15b	Amp^r, 6xHis	Novagen
pET15b all3315	6xHis-Cis12, Amp^r	This study
pET15b sfGFP-all3315	6xHis-sfGFP-Cis12, Amp^r	This study
pET15b sfGFP-all3315 ΔAH	6xHis-sfGFP-Cis12-ΔAH, Amp^r	This study
pET15b all3316	6xHis-Cis13, Amp^r	This study
pET15b sfGFP-all3316	6xHis-sfGFP-Cis13, Amp^r	This study
pET15b sfGFP-all3316 Δtmh	6xHis-sfGFP-Cis13-Δtmh, Amp^r	This study
pRL277	Integrative vector, *sacB*, Sm^r, Sp^r	Black et al., 1993[38]
pIM612	pRL1049:C.K3t4 (C.K3 cassette with transcriptional terminator), Km^r, Sm^r, Sp^r	Bornikoel, 2018[36]

pIM713	P_{all3327}-*all3317*-5xGS-sfGFP in pIM612, Km^r, Sm^r, Sp^r	This study
pIM714	Internal fragment of *all3325* in pRL277, Sm^r, Sp^r	This study
pIM815	C.K3 cassette flanked by fragments for *all3316* truncation in pRL277, Sm^r, Sp^r, Km^r	This study

Supplementary table 6: SPA parameters.

**Map**	**Baseplate C6**	**Baseplate C3**	**Crown**	**Connectors**	**Cap**	**Contracted**
Micrographs	18722	18722	18722	18722	15294	15294
Particles (initial)	228163	156948	470844	470844	433028	9780
Particles (final)	78474	36909	288285	233587	22598	3240
Pixel size (Å)	1.1	1.1	1.1	1.1	1.1	1.1
Defocus range (µm)	0.9 – 1.5	0.9 – 1.5	0.9 – 1.5	0.9 – 1.5	0.9 – 1.5	0.9 – 1.5
Voltage (kV)	300	300	300	300	300	300
Electron dose (e/Å^2)	52	52	52	52	52	52
Symmetry imposed	C6	C3	C1	C1	C6	C6
Resolution (Å) (FSC = 0.143)	2.9	3.2	2.9	3.0	2.8	6.7
Resolution range (Å)	2.6-8.4	2.8-9.9	2.8-13.4	2.8-10.4	2.6-5.4	4.3-33.0
B factor (Å^2) for map sharpening	-108	-102	-108	-118	-87	-205
EMDB	12030	12029	12032	12031	12034	12033

Supplementary table 7: Atomic model statistics.

**Map**	Baseplate C3	Cap
**EMDB**	EMD-12029	EMD-12034
**Refinement**	Baseplate	Cap
$CC_{map\text{-}model}$	0.76	0.84
**Model quality**		
RMSD		
Bond length (Å) / Bond angles (°)	0.006 / 0.786	0.006 / 0.793
Ramachandran		
Favored (%)	97.08	96.29
Outliers (%)	0	0
Rotamer outliers (%)	0	0
C-beta outliers (%)	0	0
Clashscore	3.96	4.79
MolProbity score	1.34	1.50
**PDB**	7B5H	7B5I

Supplementary table 8: Coverage and cross correlation with respective maps of all chains of the atomic model.

Baseplate model (PDB 7B5H)								
Protein	Chain ID	Total number of aa	Number of aa built	Coverage [%]	CC Baseplate C6	CC Baseplate C3	CC Crown	CC Connectors
Cis1	AN	143	142	99.3	0.81	0.79	0.12	0.40
Cis2	AO	484	473	97.7	0.87	0.88	0.33	0.73
Cis2	AP	484	480	99.2	0.86	0.85	0.26	0.58
Cis2	AQ	484	480	99.2	0.51	0.46	0.05	0.17
Cis5	AM	167	153	91.6	0.86	0.88	0.25	0.56
Cis6	AL	50	37	74.0	0.47	0.81	0.11	0.33
Cis7	AK	234	232	99.1	0.85	0.88	0.29	0.64
Cis8	AJ	589	585	99.3	0.62	0.89	0.26	0.48
Cis9	AI	149	138	92.6	0.84	0.86	0.29	0.73
Cis11	AH	1231	1227	99.7	0.87	0.86	0.50	0.74
Cis12	AD	1335	1109	83.1	0.77	0.79	0.47	0.84
Cis13	AE	154	100	64.9	0.72	0.74	0.45	0.79
Cis13	AF	154	100	64.9	0.69	0.70	0.43	0.74
Cis13	AG	154	100	64.9	0.66	0.69	0.42	0.72
Cis19	AA	1476	729	49.4	0.62	0.62	0.84	0.56
Cis19	AB	1476	724	49.1	0.60	0.59	0.84	0.54
Cis19	AC	1476	700	47.4	0.64	0.64	0.85	0.56
Cap model (PDB 7B5I)								
Protein	Chain ID	Total number of aa	Number of aa built	Coverage [%]	CC Cap			
Cis1	AD	143	142	99.3	0.85			
Cis1	AE	143	142	99.3	0.86			
Cis2	AC	484	480	99.2	0.83			
Cis16A	AA	192	187	97.4	0.83			
Cis16B	AB	399	399	100.0	0.81			

Supplementary table 9: Primers.

**Primer**	**Sequence**
GFP-all3315-5'-ins	GCAGCCATATGCTCGAGATGCGTAAAGGCGAAGAGCTG
GFP-all3315-3'-ins	CTAATGCTCAGATACTCCGGTGCCGCGGCGCCTCCCCC TTTGTACAGTTCATCCATACC
GFP-all3315-5'-bb	CCGGAGTATCTGAGCATTAG
GFP-all3315-3'-bb	CATCTCGAGCATATGGCTG
GFP-all3316-5'-ins	GCAGCCATATGCTCGAGATGCGTAAAGGCGAAGAGCTG
GFP-all3316-3'-ins	TCGTTACGCTTACGCAGGTTTGCCGCGGCGCCTCCCCC TTTGTACAGTTCATCCATACC
GFP-all3316-5'-bb	AACCTGCGTAAGCGTAACGAGCTG
GFP-all3316-3'-bb	CATCTCGAGCATATGGCTG
all3315d-5'	AGCAACGATGAGGGTTTCC
all3315d-3'	GCTCAGGTGACGACGACG
all3316d-5'	TAAGGATCCGGCTGCTAA
all3316d-3'	TTGCAGACGGATTTCCATTTG
T7	TAATACGACTCACTATAGGG
T7term	TGCTAGTTATTGCTCAGCGG
1718	AGGAAACCCCCAATGAATC
1719	CTAGACGGATATCCCGCAAGAGGCCCTTTCGTCTTCAAGG AATTTTGAATTCCGCCTTGC
1720	GGATCCACTACCGGATCCACTACCTTCACCTAACTCCAAAT CATTTAATG
1721	CCAGTTGATTCATTGGGGGTTTCCTATGAGCAACAACCGCG ATCTAC
1289	AAAGCTTATCGATGATAAGCTGTCAAACATGAGCGCGTGC TTATTTATATAATTCATCCATAC
1444	TAGTGGATCCGGTAGTGGATCCGGTAGCGCATCAAAAGGT GAAGAATTATTTAC
1722	TGATAATAAGCGGATGAATGGCAGAAATTCGATATCTAGA TCCAAATTTCCTCCCTCCATTG
1723	TAGTTCGCCAGTTAATAGTTTGCGCAACGTTGTTGCCATTG CCCACCATTGGCAGGATTG

1717	CCAGTTGATTCATTGGGGGTTTCCTATGCCAAGTACTTACAAAACTCC
890	ACCTATCTCAGCGATCTGTC
1716	GATCCACTACCGGATCCACTACCACTTTCCTGCATCTTGTGAG
2311	GATGAATGGCAGAAATTCGATATCTAGATCTCGAGGGCAATGATTAAAGTGG
2312	TGCAATCCATCTTGTTCAATCATGCGAAATCAATGTTGAAGCTTATCTAC
2313	CTTCTTGACGAGTTCTTCTGAAATTTTTAAACACAAACTAGC
2314	AATAGTTTGCGCAACGTTGTTGCCATTGCTGCACATCTTCCGCATCAGC
1864	CGTTTCGCATGATTGAACAAG
2319	TCAGAAGAACTCGTCAAG
2315	CGCCATAAAGGACGAG
2316	GAAGGTCTACCAGCAAAG

144

Supplementary Movies

Movie 1: Cryo-electron tomogram of cryoFIB-milled *Anabaena* filament (corresponds to tomogram shown in Fig. 1c/d). The cryo-tomogram is followed by a segmentation including thylakoid membranes (green), cytoplasmic membrane (dark brown), peptidoglycan (grey), storage granules (brown), cyanophycin granule (purple) and subtomogram averages of CISs (light brown). One CIS is shown as high-resolution SPA structure (color code according to Fig. 2). The SPA structure is further fitted in the *in situ* subtomogram average, revealing the identities of the narrow and wide TM-connectors.

Movie 2: Time-lapse fLM movie of UV-treated *Anabaena* Cis11-sfGFP filament showed ghost cell release (corresponds to Extended Data Fig. 7). Red fluorescence (shown in magenta) is caused by chlorophyll. The unspecific green autofluorescence is normally superimposed by red chlorophyll fluorescence in viable cells and its observation has therefore been described as marker for dead cells. T = 0 min indicates the start of fLM acquisition ~15 min after UV treatment. Imaging was continued for 1 h with an image every 2 min. Bar, 10 µm.

Movie 3: Cryo-electron tomogram of cryoFIB-milled ghost cell, followed by segmentation (corresponds to tomogram shown in Fig. 4c/d). Extended (brown) and also contracted CISs (red) were still found anchored to the outermost TM (green) and exposed to the environment. Bar, 100 nm.

Movie 4: Merged brightfield and fLM time-lapse of UV-treated *Anabaena* wild-type and CIS^{-} mutant. Both strains were able to release ghost cells upon stress (UV-treatment). Time stamp indicates time after UV-treatment. The movie was acquired over a time period of 3 h, with an image every 5 min. Bar, 10 µm.

Supplementary Source Data

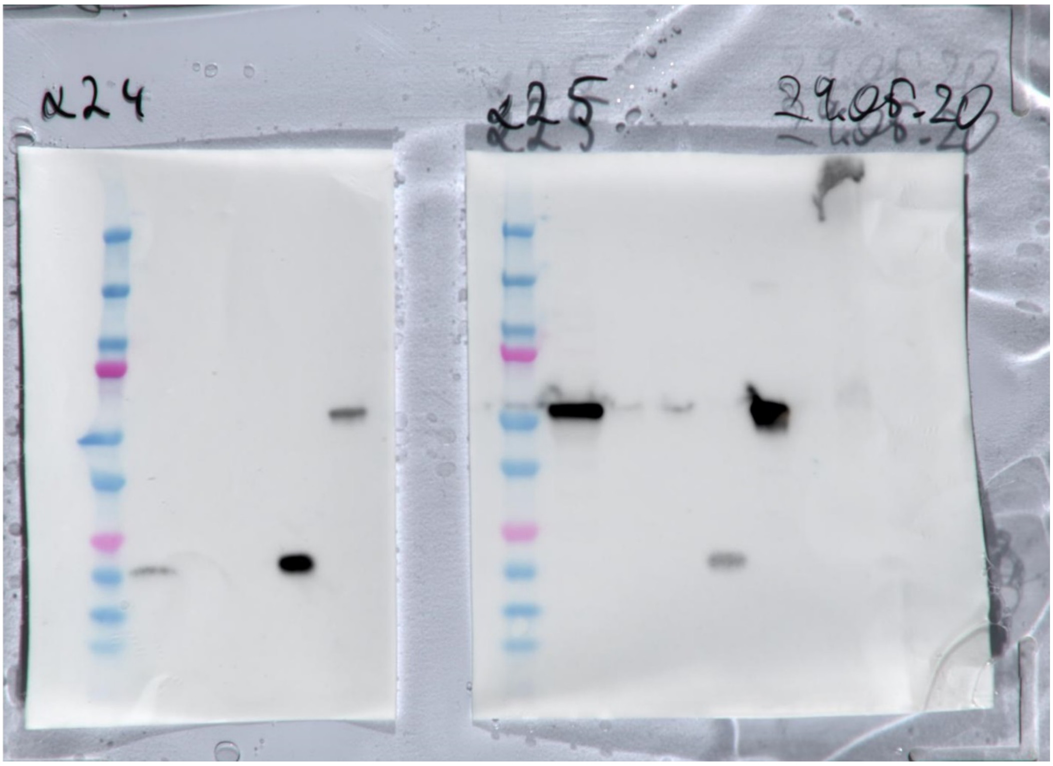

SI figure 1c: Raw image of shown western blots.

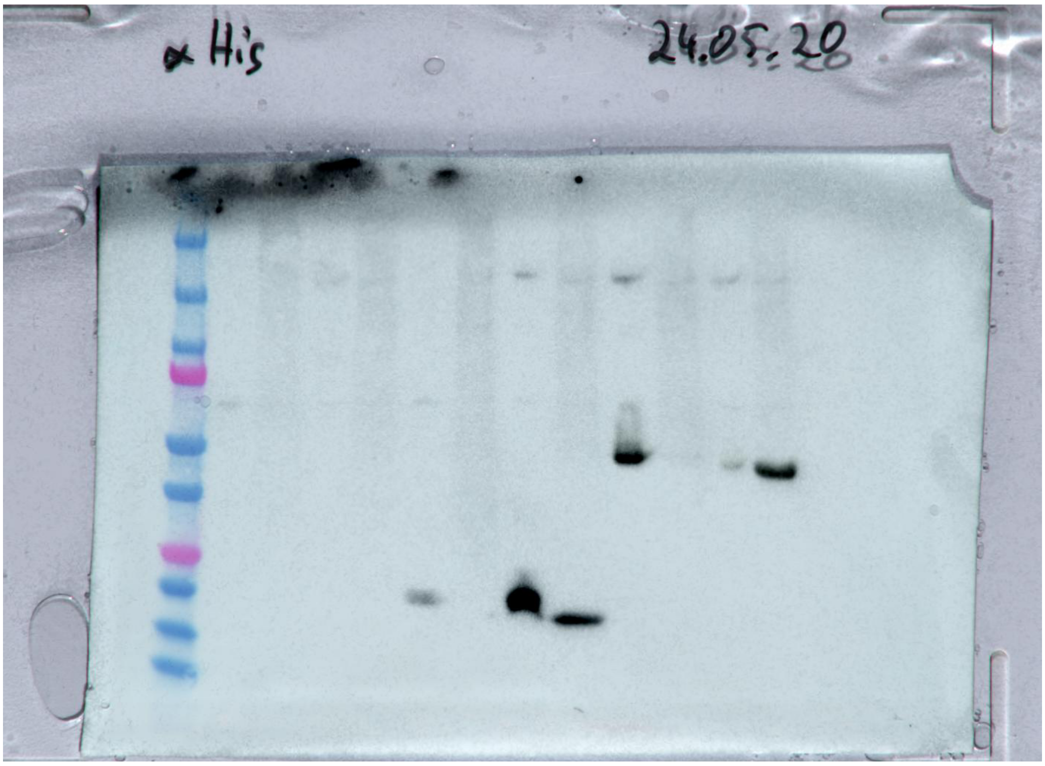

SI figure 13c: Raw image of shown western blot.
