# Supplementary material for: Structure of a thylakoid-anchored contractile injection system in multicellular cyanobacteria
Source: Nat Microbiol. 2022 Feb 14;7(3):386–96. doi: 10.1038/s41564-021-01055-y (PMC8894136; doi:10.1038/s41564-021-01055-y)
Supplement: Supplementary file 2 — Reporting Summary [file 41564_2021_1055_MOESM2_ESM.pdf]

nature research

Corresponding author(s): Pilhofer, Martin

Last updated by author(s): Dec 15, 2021

Reporting Summary

Nature Research wishes to improve the reproducibility of the work that we publish. This form provides structure for consistency and transparency in reporting. For further information on Nature Research policies, see our Editorial Policies and the Editorial Policy Checklist.

Statistics

For all statistical analyses, confirm that the following items are present in the figure legend, table legend, main text, or Methods section.

n/a	Confirmed	
☐	☒	The exact sample size (*n*) for each experimental group/condition, given as a discrete number and unit of measurement
☐	☒	A statement on whether measurements were taken from distinct samples or whether the same sample was measured repeatedly
☒	☐	The statistical test(s) used AND whether they are one- or two-sided<br>*Only common tests should be described solely by name; describe more complex techniques in the Methods section.*
☒	☐	A description of all covariates tested
☒	☐	A description of any assumptions or corrections, such as tests of normality and adjustment for multiple comparisons
☒	☐	A full description of the statistical parameters including central tendency (e.g. means) or other basic estimates (e.g. regression coefficient) AND variation (e.g. standard deviation) or associated estimates of uncertainty (e.g. confidence intervals)
☒	☐	For null hypothesis testing, the test statistic (e.g. *F*, *t*, *r*) with confidence intervals, effect sizes, degrees of freedom and *P* value noted<br>*Give P values as exact values whenever suitable.*
☒	☐	For Bayesian analysis, information on the choice of priors and Markov chain Monte Carlo settings
☒	☐	For hierarchical and complex designs, identification of the appropriate level for tests and full reporting of outcomes
☒	☐	Estimates of effect sizes (e.g. Cohen's *d*, Pearson's *r*), indicating how they were calculated

Our web collection on statistics for biologists contains articles on many of the points above.

Software and code

Policy information about availability of computer code

Data collection	SPA cryoEM and cryoET data collection: SerialEM 3.7<br>cryoFIB milling: Thermo Fisher Scientific XT software<br>Light microscopy: Visiview, Zen, LasX, Fiji
Data analysis	cryoET and subtomogram averaging: IMOD 4.11, PEET, Dynamo, Chimera<br>single-particle cryoEM: IMOD 4.11, RELION 3.0, crYOLO, COOT, RosettaCM, PHENIX, Chimera, gctf, MolProbity, mtriage, ChimeraX

For manuscripts utilizing custom algorithms or software that are central to the research but not yet described in published literature, software must be made available to editors and reviewers. We strongly encourage code deposition in a community repository (e.g. GitHub). See the Nature Research guidelines for submitting code & software for further information.

Data

Policy information about availability of data

All manuscripts must include a data availability statement. This statement should provide the following information, where applicable:

- Accession codes, unique identifiers, or web links for publicly available datasets
- A list of figures that have associated raw data
- A description of any restrictions on data availability

Example tomograms (EMD-13770 - EMD-13772), subtomogram averages (EMD-13773 – EMD-13775) and SPA cryoEM maps (EMD-12029 – EMD-12034, see Table S6) were uploaded to the Electron Microscopy Data Bank. Atomic coordinates of the baseplate (PDB 7B5H) and cap complex (PDB 7B5I) have been uploaded to the Protein Data Bank.

Other datasets used in this study from the Protein Data Bank (PDB): 6J0F, 6RAP, 6J0B, 6RBN, 6J0N, 6RBN, 6RAO

Field-specific reporting

Please select the one below that is the best fit for your research. If you are not sure, read the appropriate sections before making your selection.

☒ Life sciences ☐ Behavioural & social sciences ☐ Ecological, evolutionary & environmental sciences

For a reference copy of the document with all sections, see nature.com/documents/nr-reporting-summary-flat.pdf

Life sciences study design

All studies must disclose on these points even when the disclosure is negative.

Sample size	All particles (n = 209) found in 99 high-quality tomograms were used for initial in situ subtomogram average of extended CIS. Final particle number after corss-correlation cleaning: 170.<br>All particles (n =78) found in 15 high-quality tomograms were used for initial subtomogram average of CIS in ghost cells. FInal particle number after CC cleaning: 64.<br>All particles (n = 11) found in 6 high-quality tomograms were used for subtomogram average of contracted CIS.<br>For SPA cryoEM, please see Fig. S5 for detailed numbers of particles as well as Methods section.<br><br>For all other experiments, no sample size determination was performed.
Data exclusions	Particles which were not fully inside the field of view were excluded. CISs seen in tomograms of bad quality were excluded.
Replication	Replication of cryoEM and cryoET findings was not attempted.<br>Replications of light microscopy findings were successful at all attempts and the number of replications are stated in figure legends.
Randomization	Extracted particles (for SPA and subtomogram averaging) were randomly assigned to two separate groups to calculate half-maps and gold-standard FSC.<br>For other experiments, no randomization was performed.
Blinding	CryoEM projection images of ghost cells released from wild-type and CIS deficient mutant were anonymized and blindly sorted into classes ("CM intact" and "CM ruptured/absent"). For all other experiments, blinding was not attempted.

Reporting for specific materials, systems and methods

We require information from authors about some types of materials, experimental systems and methods used in many studies. Here, indicate whether each material, system or method listed is relevant to your study. If you are not sure if a list item applies to your research, read the appropriate section before selecting a response.

Materials & experimental systems

n/a	Involved in the study	
☐	☒	Antibodies
☐	☒	Eukaryotic cell lines
☒	☐	Palaeontology and archaeology
☐	☒	Animals and other organisms
☒	☐	Human research participants
☒	☐	Clinical data
☒	☐	Dual use research of concern

Methods

n/a	Involved in the study	
☒	☐	ChIP-seq
☒	☐	Flow cytometry
☒	☐	MRI-based neuroimaging

Antibodies

Antibodies used	For His-tagged samples, membranes were incubated with 1:5000 HRP-conjugated anti-6xHis antibody (#MA1-21315-HRP, Invitrogen). Alternatively, membranes were incubated with 1:1000 polyclonal rabbit anti-all3324 (anti-Cis1) or anti-all3325 (anti-Cis2) antibody (GenScript) and 1:5000 secondary horseradish peroxidase-conjugated goat anti-rabbit IgG (Abcam).
Validation	Validation of antibodies was done by manufacturer (SDS-PAGE of Antigen and Western Blot, Elisa) as well as with western blotting against Anabaena wild-type and CIS deficient mutant.

Eukaryotic cell lines

Policy information about cell lines

Cell line source(s)	Sf9 insect cells

Authentication	None of the used cell lines were authenticated.
Mycoplasma contamination	Cell lines were not tested for mycoplasma contamination.
Commonly misidentified lines (See ICLAC register)	*Name any commonly misidentified cell lines used in the study and provide a rationale for their use.*

Animals and other organisms

Policy information about studies involving animals; ARRIVE guidelines recommended for reporting animal research

Laboratory animals	Ciliates, Daphniae, Hydroides elegans tubeworm larvae, wax moth larvae
Wild animals	No wild animals haven been used in this study.
Field-collected samples	Water samples from Lake Zürich were collected at the center of the lake in front of Thalwil, using a twin plankton net (2x 100 µm mesh) tow from a depth of 10 m to the surface. 200 mL of this sample was then stored at 4 °C and used for CIS purification.
Ethics oversight	No ethics approval was required.

Note that full information on the approval of the study protocol must also be provided in the manuscript.
